# L-carvone supplementation reduces methane production and modulates rumen fermentation, digestibility and microbial communities *in vitro*

**DOI:** 10.3389/fvets.2026.1900394

**Published:** 2026-07-31

**Authors:** Hassan Jalal, Luigi Pompei, Melania Giammarco, Muhammad Irfan Malik, Shazia Akram, Muhammad Zeeshan Akram, Marco Di Domenico, Francesca Ghiaccio, Riccardo Colleluori, Paolo Pezzi, Isa Fusaro

**Affiliations:** 1 Department of Veterinary Medicine, University of Teramo, Teramo, Italy; 2 Department of Animal Science, The University of Tennessee Institute of Agriculture (UTIA), Knoxville, TN, United States; 3 Department of Bioscience and Technology for Food, Agriculture and Environment, University of Teramo, Teramo, Italy; 4 Nutrition and Animal-Microbiota Ecosystems Laboratory, Department of Biosystems, KU Leuven, Heverlee, Belgium; 5 Istituto Zooprofilattico Sperimentale dell’Abruzzo e del Molise “G. Caporale”, Teramo, Italy; 6 Department of Veterinary Medical Sciences, University of Bologna, Bologna, Italy,

**Keywords:** digestibility, essential oils, L-carvone, methane, rumen microbiome, volatile fatty acids

## Abstract

**Introduction:**

Natural feed additives are increasingly explored to reduce ruminal methane emissions. This study evaluated L-carvone (LC), a monoterpene compound present in essential oils, for its effects at different inclusion levels on rumen fermentation, *in vitro* dry matter digestibility (IVDMD), methane production, and microbiota composition.

**Methods:**

Three treatments were tested: a basal diet (total mixed ration; TMR + 0 μL/L LC), LC250 (TMR + 250 μL/L LC), and LC500 (TMR + 500 μL/L LC), using a 59:41 concentrate-to-forage substrate. Fermentation parameters were analysed using the Gas Endeavour system and 16 S rRNA gene sequencing was applied to investigate the ruminal microbiota profile. Data were analysed using linear mixed models, with treatment included as a fixed effect and experimental runs as a random effect. Pairwise comparisons were conducted using Bonferroni correction, with *p* value below 0.05 considered significant.

**Results:**

Total gas production decreased progressively with increasing LC inclusion (*p <* 0.001), whereas methane production was significantly reduced only in LC500 (*p* < 0.001). LC500 reduced methane production by 28% compared with TMR (*p <* 0.001) and decreased IVDMD by approximately 15%, while LC250 showed a numerical but non-significant reduction in methane production without affecting IVDMD. Total volatile fatty acid (TVFA) concentration was lower in LC500compared with TMR (*p* < 0.01), whereas LC250 showed intermediate values. This reduction was mainly due to lower propionate, iso-butyrate, and iso-valerate concentrations. The proportion of butyrate increased in LC500 (*p* < 0.01). In addition, ammonia nitrogen concentration was lower in LC500 than in LC250 (*p* = 0.040), while TMR remained intermediate. At the phylum level, increasing LC supplementation reduced the abundance of Bacteroidota and increased Firmicutes. At the genus level, *Prevotella* abundance decreased with LC inclusion, whereas butyrate-producing genera including *Butyrivibrio, Pseudobutyrivibrio,* and *Ruminococcus* increased. Alpha diversity analysis showed that richness indices (observed OTUs and Faith’s phylogenetic diversity) were highest in LC250, whereas Shannon diversity and Pielou’s evenness were similarly higher in LC250 and LC500 compared with TMR.

**Conclusion:**

The LC reduced methane production during a 24-h *in vitro* rumen fermentation while altering rumen fermentation characteristics, as evidenced by lower total gas production, IVDMD, and VFA concentrations at the highest inclusion level. These changes were accompanied by shifts in the rumen microbiota, including a reduced abundance of Euryarchaeota, a phylum associated with methanogenic archaea. Therefore, optimizing the inclusion level of LC is essential to achieve methane mitigation while minimizing adverse effects on feed digestion.

## 1 Introduction

Climate change, driven by anthropogenic activities, poses an escalating threat to global ecosystems and food security, which is worsened by the growing human population and its demand for animal-derived protein (1). Ruminants are essential for fulfilling this demand but significantly contribute to greenhouse gas (GHG) emissions, primarily through enteric methane (CH_4_) production (2). The livestock sector accounts for approximately 14.5% of total anthropogenic GHG emissions, with enteric fermentation alone contributing nearly 40% of agricultural GHG emissions and resulting in a 3–12% loss of gross energy intake in ruminants (3). Reducing enteric CH_4_ emissions while enhancing ruminant productivity is hence a dual necessity for sustainable agriculture. Ionophores, though effective in optimizing rumen fermentation, have been phased out in regions such as the European Union since January 2006 under Regulation (EC) No 1831/2003 (4, 5), due to concerns regarding antimicrobial resistance and consumer safety.

Consequently, substantial research efforts have been directed toward in evaluating other alternatives strategies to modulate rumen fermentation, such as yeasts, plant derived bioactive compounds, organic acids and probiotics (6). Plant extracts or plants derived bioactive compounds, such as essential oils (EOs) and their constituents, offer a promising alternative as natural rumen modifiers (7). These compounds possess various functional groups, including aliphatic hydrocarbons, aldehydes, acids, esters, ketones, alcohols and epoxides (8), enabling EOs to interact with multiple molecular and cellular targets to induce targeted physiological and biochemical responses (9). These secondary metabolites modulate microbial activity, reduce CH_4_ production, and enhance nutrient utilization (10). EOs have been regarded as a promising option for the development of natural additives that can modulate rumen activity in a manner more compatible with the rumen ecosystem, owing to their antimicrobial properties (11). Importantly, the composition of EOs changes due to a variety of factors like the phenological stage or section of the plant, geographic position, and extraction methods, which require focused investigations on specific bioactive substances. Consequently, an accurate characterization of the effects induced by the various active compounds present in EOs is necessary to fully exploit their potential health benefits, nutritional advantages, or environmental impacts when incorporated into the diets of ruminants as CH_4_ mitigating additive. While significant research has focused on phenolic monoterpenes (e.g., carvacrol, thymol, eugenol), alcoholic monoterpenes (e.g., linalool, geraniol), and hydrocarbon monoterpenes (e.g., limonene, *γ*-terpinene), while monoterpene ketones such as L-carvone (LC) remain underexplored, particularly in the context of ruminant fermentation research (12). Numerous studies reveal that high levels of thymol and carvacrol (> 225 mg/L), while partially suppressing rumen methanogenesis, may negatively impair rumen fermentation, which is nutritionally detrimental to the host animal (13). Because of their low molecular weight, these substances may facilitate their gradual diffusion across the cell wall through the lipopolysaccharide layer or membrane proteins, enabling interaction with the lipid bilayer of cells, hence diminishing their selectivity toward certain microbial groups (5). Conversely, compounds exhibiting minimal antimicrobial activity, like *p*-cymene, diminish methanogenic activity at low concentrations (20 mg/L) without negatively impacting ruminal fermentation, likely due to their more targeted effects relative to thymol or carvacrol on rumen methanogens (11).

Therefore, further systematic research is required to assess the impact of pure bioactive compounds of EOs on animal nutrition. Among these bioactive compounds, LC is characterized by a complex chemical structure and exhibits antimicrobial potential (14); however information regarding its effects on rumen fermentation and CH_4_ production remains limited. The scarce research on LC’s influence on rumen fermentation dynamics and microbial communities reveals a significant knowledge gap, underscoring the necessity to conduct further research. Therefore, this study evaluated whether LC, a monoterpene ketone present in caraway (*Carum carvi*) and spearmint (*Mentha spicata*), could mitigate CH_4_ production while modulating rumen fermentation characteristics and microbial community composition in an *in vitro* fermentation system.

## 2 Materials and methods

All animal handling procedures were conducted in accordance with institutional guidelines and approved by the Scientific Ethics Committee of the Faculty of Veterinary Medicine, University of Teramo (Prot. n. 18528, 28/06/2022).

### 2.1 Chemical composition analysis

The ingredients and chemical composition of the total mixed ration (TMR) used as the basal diet are presented in Table 1. Except stated otherwise, all chemical analyses were performed using AOAC method (15). The dry matter (DM) content was determined by oven-drying samples at 100 °C for 12 h, crude protein (CP) was quantified using a Kjeltec 8,100 analyzer (AOAC Method 984.13), ether extract (EE) via Soxtec Avanti solvent extraction (AOAC 920.39), and ash content via incineration in a muffle furnace at 550 °C (AOAC 942.05). The neutral detergent fibre (NDF), acid detergent fibre (ADF), acid detergent lignin (ADL) were analysed sequentially using an FT 122 Fibertec analyzer. The ash-corrected NDF analysis was carried out using heat-resistant *α*-amylase pretreatment, following the methodology of Van Soest et al. (16).

**Table 1:** Ingredient and chemical composition of the total mixed ration.

Items	g/kg of DM (unless otherwise stated)
Ingredient	
Corn silage	134.3
Alfalfa hay	129.6
Sorghum silage	53.7
Triticale silage	19.2
Grass hay	57.1
Wheat straw	14.7
Ground corn	187.0
Soybean meal 48%	115.1
Steam-flaked corn	83.7
Wheat middlings	41.9
Whole cottonseed	58.6
Liquid sugar supplement	28.9
Ground barley	28.4
Corn gluten feed	14.7
Calcium soap (protected fat)	7.6
Mineral premix	15.8
Sodium bicarbonate	7.9
Chemical composition	
Dry matter g/kg of fresh matter	564
Crude protein	141
Neutral detergent fibre	322
Acid detergent fibre	188
Acid detergent lignin	40
Ether extract	28
Ash	74

### 2.2 Experimental design and treatment

The bioactive compound LC (purity ≥ 99.0%; specific gravity 0.955–0.966 g/mL) was obtained from Shree Bankey Behari Lal Aromatics (Uttar Pradesh, India) and used in the present *in vitro* study. The compound was stored in a cool, dry, and dark place away from direct sunlight according to the supplier’s recommendations.

The experiment comprised six independent *in vitro* fermentations, evaluating three treatments using a substrate with a 59:41 concentrate-to-forage ratio (14.1% CP, 32.2% NDF), which served as the basal diet: TMR (0 μL/L LC), LC250 (TMR + 250 μL/L LC), and LC500 (TMR + 500 μL/L LC). The inclusion levels of LC (250 and 500 μL/L) were selected based on previous *in vitro* studies evaluating EOs bioactive compounds within a similar concentration range (12, 17, 18).

Of the six independent fermentations, three were performed for total gas production measurements, and three separate fermentations were performed for CH_4_ quantification. In each fermentation run, each treatment was represented by four incubation bottle replicates. Consequently, each treatment was represented by 12 replicates for gas production across three fermentation runs (4 replicates × 3 runs; *n* = 12) and 12 replicates for CH_4_ production (4 replicates × 3 runs; *n* = 12). Each fermentation run was conducted using freshly collected rumen fluid on different days and therefore represented an independent fermentation batch. Samples used for VFA, NH_3_-N, and microbiota analyses were collected from the CH_4_ fermentation runs. In addition, one negative control bottle containing buffered rumen fluid without substrate was included in each fermentation run to correct gas and CH_4_ production values for background production.

### 2.3 Ruminal inoculum preparation

Rumen fluid used in this study was collected from three healthy lactating cows using an esophageal tube following the standard, non-invasive procedures. The donor cows were fed ad libitum a TMR consisting primarily of corn silage, alfalfa hay and a concentrate mixture at a 59:41 concentrate-to-forage ratio. Rumen fluid was collected before the morning feeding (07:00 h) and equal volume from three donor cows were pooled prior to incubation. For each experimental run, fresh rumen fluid was collected before incubation from the donor cows. A pre-warmed thermos kept at 39 °C was instantly filled with the collected rumen fluid, which was around 2,000 mL in volume and taken to the laboratory without being delayed. The rumen fluid was filtered through four layers of muslin cloth and transferred to an Erlenmeyer flask maintained at 39 °C with continuous carbon dioxide (CO_2_) purging. The buffer mixture for *in vitro* rumen fermentation was prepared according to the Goering and Soest (19). Briefly, fermentation medium was made using deionised H_2_O, a buffer solution (consisting of NH_4_HCO_3_, NaHCO_3_, deionised H_2_O), a macromineral solution (consisting of Na_2_HPO_4_, KH_2_PO_4_, and MgSO_4_.7H_2_O), a micromineral solution (consisting of CaCl_2_.2H_2_O, CoCl_2_.6H_2_O, MnCl_2_.4H_2_O, and FeCl_3_.6H_2_O). Reducing agents, including sodium sulphide anhydrous and L-cysteine hydrochloride, were added 15 min before transferring the flasks to the water bath under CO_2_ conditions.

### 2.4 Gas production measurement

Total Gas and CH_4_ production during *in vitro* incubation were determined using the Gas Endeavour System (GES). The GES (BPC Instruments, Sweden), is an automated volumetric gas flow measurement system designed to detect low gas volumes (20). Gas production is quantified based on the liquid displacement and buoyancy principles (21). This apparatus facilitates the simultaneous analysis of multiple samples within a temperature-regulated water bath containing 15 fermentation bottles, continuously agitated at a specified rpm. The water bath functions as an incubator, sustaining a stable temperature of 39 °C using an automatic heating controller. For fermentation, 5.0 ± 0.1 g of TMR was added to 500 mL Duran bottles, followed by 400 mL of buffered medium, with LC added according to the experimental treatments. After pre-incubation under CO_2_ for 10–15 min, 100 mL of filtered rumen fluid was added to each bottle. The bottles were sealed, placed in a thermostatically controlled water bath at 39.5 °C. For CH_4_ quantification, the fermentation bottles were connected to CO_2_ absorption unit, which was filled with 3 M NaOH to selectively remove CO_2_ and N_2_O and allowing only CH_4_ to pass to the gas sensors. Each incubation run was conducted over a 24-h period, with real-time gas production measurements gathered and displayed via the system’s connected software.

### 2.5 Analysis of fermentation parameters

Following 24 h of incubation, rumen fluid samples from each fermentation bottle were filtered using glass crucibles, and the filtrate was subsequently transferred to 20 mL centrifuge tubes for pH analysis. Aliquots were preserved at −20 °C for further study of VFAs, NH_3_-N, and for DNA extraction. Before analysis of VFAs and NH_3_-N, rumen fluid samples were thawed and centrifuged at 10,000×*g* for 10 min to remove particulate matter and clarify the sample. VFAs were analysed by gas chromatography according to the method of Goetsch et al. (22), using an HRGC MEGA 2 series gas chromatograph (Mega Gas Chromatography Solutions, Legnano, Italy). Individual VFAs were identified using external standards, and concentrations were quantified using 2-Ethylbutyric acid (Sigma-Aldrich, St. Louis, MO) as an internal standard and expressed as mmol/L. The NH_3_-N concentration was assessed utilising an enzymatic colorimetric assay in accordance with the manufacturer’s instructions (Urea/BUN-Color; BioSystems S. A., Barcelona, Spain). Absorbance was measured using an Ultrospec 3,000 spectrophotometer (Biochrom Ltd., Cambridge, UK) at 532 and 600 nm. The final NH_3_-N concentrations were expressed in mg/dL.

### 2.6 *In vitro* dry matter digestibility

The IVDMD of TMR supplemented with LC at different doses was evaluated using the Daisy II Incubator (Ankom Technology, USA), following the method of Van Soest et al. (16). The buffered medium was diluted 1:4 (v/v) with fresh rumen fluid, which was collected and processed as described in previous section. Two liters of buffered rumen fluid medium were anaerobically transferred to 5-liter incubation jars equipped with one-way valves to inhibit gas buildup. For each treatment, three incubation jars were used, and LC was added to the incubation jars at the designated concentrations (0, 250, and 500 μL/L), based on the total volume of buffered rumen fluid medium (2 L per jar). Each incubation jar contained 21 substrate bags (250 mg TMR per bag) and three blank bags. To establish anaerobic conditions, jars were purged with CO_2_ and then incubated at 39 °C in a rotating Daisy II Incubator to assure that the bags were continuously exposed in the rumen fluid medium. Bags were rinsed with cold water after incubation, dried for 12 h at 100 °C, and weighed. IVDMD was calculated based on the difference between the substrate weight incubated and the oven-dried residue remaining after incubation, as follows:

IVDMD(%)=[100−((W3−(W1×C1))/W2)×100)]

where W1 = empty bag weight, W2 = initial substrate weight, W3 = final oven-dried bag weight after *in vitro* incubation, and C1 = blank bag correction (final oven-dried weight/original blank bag weight).

### 2.7 DNA extraction, PCR amplification, and 16S rRNA sequencing

Rumen fluid samples were thawed at ice for 30 min and immediately centrifuged at 10.000 rpm for 10 min at room temperature. Supernatants were discarded, and pellets were resuspended in phosphate-buffered saline (23). DNA was extracted from the pellets using an E.Z.N.A.™ stool DNA isolation kit (Omega Bio-Tek) following the manufacturer’s instructions. DNA concentration was measured using the QuantiFluor® ONE dsDNA System Kit and Quantus™ Fluorometer (Promega Corporation, Madison, WI) following the manufacturer’s guideline. NanoDrop Spectrophotometer (Thermo Fisher Scientific, Waltham, MA, USA), was used to access the purity of DNA samples. Nimagen EasySeq™ 16S Microbiome Library Prep Kit (Nimagen B.V., Nijmegen, The Netherlands), was used to create amplicon libraries that targets the16S rRNA gene. This kit powered by Reverse Complement PCR (RC-PCR), provides a one-tube single reaction workflow to characterize the microbiota through amplification of the hypervariable regions V3-V4, using the forward 5′-CCTACGGGNGGCWGCAG-3′ and reverse 5′-GACTACHVGGGTATCTAATCC-3′primers. PCR products were pooled in a single tube and purified using AmpliClean Cleanup Kit (Nimagen B. V., Nijmegen, The Netherlands). The amplicon libraries were subjected to quality control to verify the size distribution and concentration using an Agilent TapeStation 4200 System (Agilent Technologies, Santa Clara, CA) with Agilent D5000 ScreenTape reagents and the Qubit 2.0 Fluorometer (Thermo Fisher Scientific, Waltham, MA), respectively. Sequencing was run on an Illumina NextSeq1000 using 600 cycles (2 × 300) NextSeq 1000/2000 P1 XLEAP-SBS Reagents and P1 cartridge. Fastq files were processed using the Genpat platform, the Italian National Reference Centre for WGS of microbial pathogens.^1^ Briefly, raw reads were normalised at 500 K reads (24) and then processed using nf-core/ampliseq v2.12.0^2^ (25) of the nf-core collection of workflows (26), utilising reproducible software environments from the Bioconda (27) and Biocontainers (28) projects. The pipeline was executed with Nextflow v23.04.2 (29).

### 2.8 Statistical analysis

Statistical analyses for fermentation parameters and IVDMD were performed using SPSS Version 29.0.1.0. Normality of data was confirmed via the Shapiro–Wilk test. Fermentation parameters were analysed using a linear mixed model with treatment (TMR, LC250, LC500) as a fixed effect and fermentation run as a random effect. Pairwise comparisons between treatments were conducted via Bonferroni correction. IVDMD data were evaluated via one-way ANOVA followed by Tukey’s *post hoc* test. Microbiota data, including relative abundances of genera, alpha diversity indices, and beta diversity, were analysed using R (v4.4.0). The Kruskal–Wallis test was used to compare alpha diversity indices (Observed OTUs, Shannon entropy, Faith’s phylogenetic diversity and Pielou’s evenness) among treatments. *p* values from all pairwise comparisons of alpha diversity were adjusted for multiple testing using the Benjamini–Hochberg false discovery rate (FDR) procedure. Principal coordinate analysis (PCoA) was performed to display beta diversity, which was calculated using Bray–Curtis and Jaccard dissimilarity matrices. Permutational multivariate analysis of variance (PERMANOVA) was applied to assess differences in the microbial community composition across treatments. The top 30 genera based on relative abundance were analysed among treatments using the Kruskal–Wallis test, followed by pairwise Wilcoxon rank-sum tests, with *p*-values adjusted for multiple comparisons using the Benjamini–Hochberg FDR method. Pairwise Pearson’s correlation coefficients (*r*) among continuous variables were calculated using the rcorr () function from the Hmisc package, which provides both correlation coefficients and corresponding *p*-values. CH_4_ was considered the primary response variable. Variables significantly correlated with CH_4_ (*p <* 0.05) were identified and ranked based on the absolute value of their correlation coefficients. To make the visualization easier to understand, non-significant correlations (*p ≥* 0.05) were removed. Plots and figures were generated by ggplot2 R package. Differences were considered statistically significant at *p* < 0.05. Data are reported as group means with pooled standard errors of the mean.

## 3 Results

### 3.1 Gas production

At all-time points, both LC treatments showed significantly (*p <* 0.001) lower gas production compared to the TMR (Table 2). However, LC250 and LC500 did not significantly differ at 2 h, whereas LC500 showed lower gas production than LC250 at the remaining time points (*p <* 0.001). The gas yield in LC500 was reduced by approximately 7.6% compared to LC250 and by 15.4% compared to the TMR at the 24th hour.

**Table 2:** Gas production (mL/g substrate DM) at different incubation times for the three dietary treatments supplemented with L-carvone.

^1^Parameters	^2^Dietary treatments			^3^SEM	*p*-value
	TMR	LC250	LC500		
2 h	15.6^a^	8.8^b^	9.1^b^	0.47	<0.001
4 h	52.7^a^	35.1^b^	32.1^c^	1.19	<0.001
6 h	87.6^a^	67.5^b^	61.4^c^	1.75	<0.001
8 h	116.8^a^	95.4^b^	87.2^c^	2.70	<0.001
10 h	138.8^a^	117.6^b^	107.1^c^	3.07	<0.001
12 h	152.9^a^	133.8^b^	121.8^c^	3.05	<0.001
16 h	166.5^a^	151.1^b^	140.1^c^	4.03	<0.001
20 h	176.8^a^	161.7^b^	150.2^c^	4.04	<0.001
24 h	185.6^a^	170.5^b^	157.1^c^	4.14	<0.001

^1^2–24 h: gas production (mL/g substrate DM) measured at 2, 4, 6, 8, 10, 12, 16, 20 and 24 h; ^2^TMR; total mixed ration = (0 μL/L of LC); LC250 = (TMR + 250 μL/L of LC); LC500 = (TMR + 500 μL/L of LC); ^3^SEM: pooled standard error of the mean. ^a–c^Means with different superscripts in a row differ significantly (*p* < 0.05).

### 3.2 Methane production

Across all time points, the LC500 treatment consistently reduced CH_4_ production compared to both TMR and LC250 (*p* < 0.01; Table 3). The CH_4_ yield in LC500 was reduced by approximately 24.1% compared to LC250 and by 28.2% compared to the TMR at 24th hour. In contrast, the reduction in CH_4_ production for LC250 compared with TMR was numerical (5.41%) rather than statistically significant.

**Table 3:** Methane production (mL/g substrate DM) at different incubation times for the three dietary treatments supplemented with L-carvone.

^1^Parameters	^2^Dietary treatments			^3^SEM	*p*-value
	TMR	LC250	LC500		
2 h	8.5^a^	7.8^a^	4.5^b^	1.10	<0.001
4 h	18.7^a^	16.8^a^	10.6^b^	1.49	<0.001
6 h	26.1^a^	23.8^a^	16.7^b^	1.87	<0.001
8 h	30.8^a^	28.6^a^	20.3^b^	2.32	<0.001
10 h	34.0^a^	31.9^a^	22.8^b^	2.71	<0.001
12 h	36.0^a^	34.0^a^	24.50^b^	3.05	<0.001
16 h	38.8^a^	37.0^a^	27.3^b^	2.95	<0.001
20 h	40.9^a^	39.0^a^	29.3^b^	3.09	<0.001
24 h	42.5^a^	40.2^a^	30.5^b^	2.90	<0.001

^1^2–24 h: methane production (mL/g substrate DM) measured at 2, 4, 6, 8, 10, 12, 16, 20 and 24 h; ^2^TMR; total mixed ration = (0 μL/L of LC); LC250 = (TMR + 250 μL/L of LC); LC500 = (TMR + 500 μL/L of LC); ^3^SEM: pooled standard error of the mean. ^a–b^Means with different superscripts in a row differ significantly (*p* < 0.05).

### 3.3 *In vitro* dry matter digestibility, pH, volatile fatty acids and ammonia nitrogen

A significant reduction in IVDMD was observed in LC500 compared with TMR and LC250 (*p* < 0.001; Table 4), whereas ruminal pH was not affected by LC supplementation (*p* = 0.301). Acetate and valerate concentration were not influenced (*p >* 0.05) by LC supplementation. Propionate and iso-butyrate were significantly lower in LC500 than in TMR, while LC250 did not differ from either group. Butyrate was higher in LC500 as compared to LC250 and TMR (*p* = 0.01), and the lowest butyrate was observed in TMR. The acetate to propionate ratio did not differ significantly among treatments (*p >* 0.05). Total VFA concentration was lower in LC500 than in TMR (*p* = 0.01), whereas LC250 was comparable to both treatments. NH_3_-N concentration was lower in LC500 than in LC250 (*p* = 0.04), while TMR did not differ from either treatment.

**Table 4:** pH, *in vitro* dry matter digestibility, molar proportion of volatile fatty acids and ammonia nitrogen concentration of the three dietary treatments supplemented with L-carvone.

^1^Parameters	^2^Dietary treatments			^3^SEM	*p*-value
	TMR	LC250	LC500		
pH	6.89	6.88	6.94	0.03	0.301
IVDMD%	77.4^a^	75.8^a^	65.6^b^	1.13	<0.001
VFA (% of total VFA)					
Acetate	53.1	53.4	52.6	0.61	0.21
Propionate	24.8^a^	24.0^ab^	23.0^b^	0.56	0.01
Iso Butyrate	1.8^a^	1.7^ab^	1.6^b^	0.10	0.02
Butyrate	13.1^c^	13.8^b^	15.7^a^	0.22	0.01
Iso Valerate	3.2^a^	3.1^a^	2.9^b^	0.18	0.01
Valerate	4.0	4.0	4.2	0.07	0.07
Acetate: Propionate	2.2	2.2	2.3	0.06	0.07
TVFA mmol/l	70.1^a^	68.7^ab^	62.6^b^	1.73	0.01
Ammonia nitrogen (mg/dl)	29.5^ab^	30.4^a^	26.8^b^	1.83	0.04

^1^TVFA = total volatile fatty acids; ^2^TMR; total mixed ration = (0 μL/L of LC); LC250 = (TMR + 250 μL/L of LC); LC500 = (TMR + 500 μL/L of LC); ^3^SEM: pooled standard error of the mean; ^a–c^Means with different superscripts in a row differ significantly (*p* < 0.05).

### 3.4 Rumen bacterial community

#### 3.4.1 Phylum-level relative abundance

**Figure 1:**
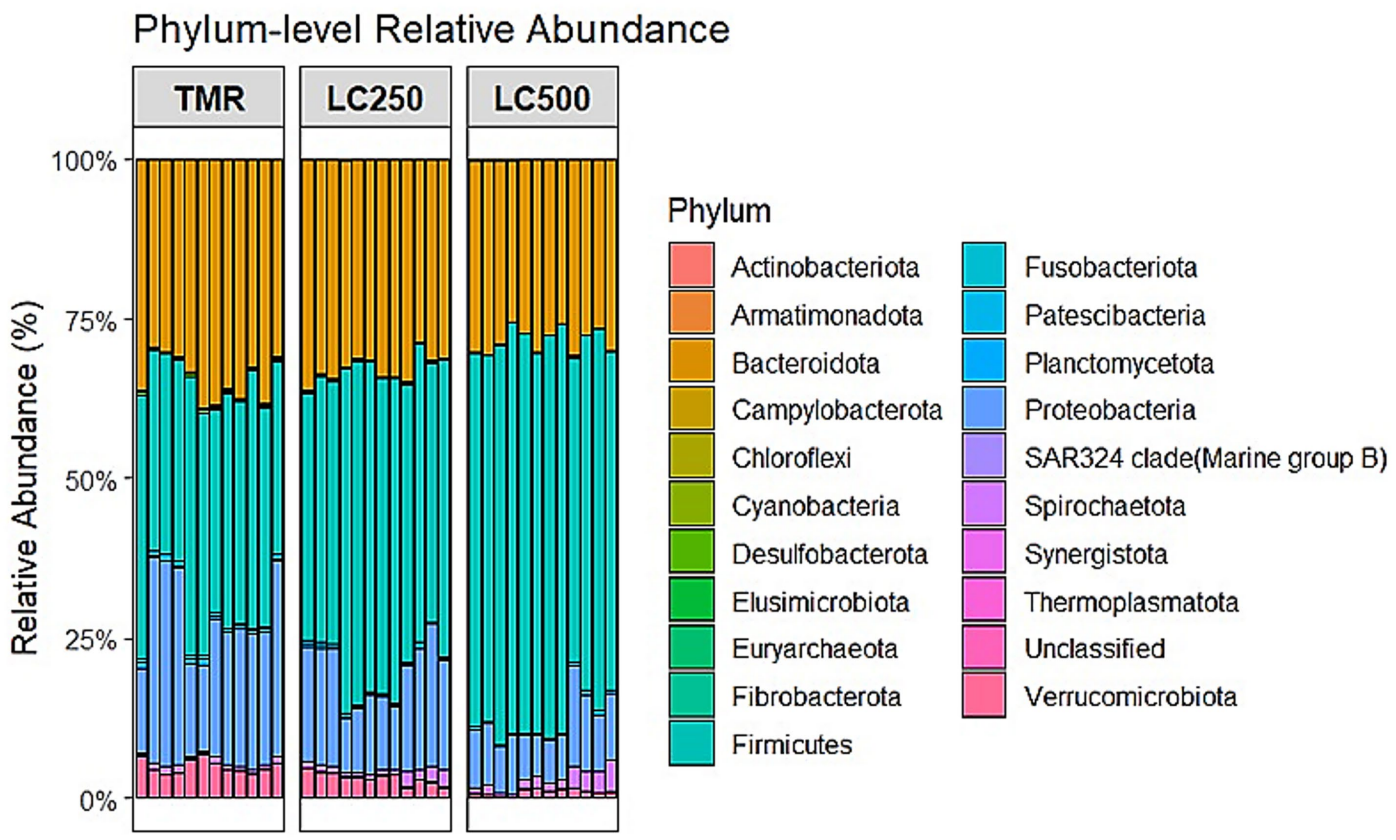
Microbial community composition at the phylum level across treatment groups. Stacked bar plots illustrating the relative abundance (%) of major phyla within the TMR (Total Mixed Ration; 0 μL/L LC), LC250 (TMR + 250 μL/L LC), and LC500 (TMR + 500 μL/L LC) treatment groups. Each bar represents a single sample, and the coloured segments indicate the proportion of each phylum. Colours correspond to the phyla listed in the legend.

The overall bacterial community structure at the phylum level exhibited clear, dose-dependent shifts in response to LC supplementation (Figure 1). Across all treatments, the rumen microbiota was primarily composed of Bacteroidota and Firmicutes, together accounting for more than 80% of total abundance, followed by Proteobacteria, Spirochaetota, Verrucomicrobiota, and Actinobacteriota, with low abundances of methanogenic Euryarchaeota. Bacteroidota was dominant in the TMR group, and its relative abundance progressively decreased with increased LC concentration. However, Firmicutes, on the other hand, rose proportionately and became the dominating phylum at the LC500 dose. Minor Phyla like Synergistota and Spirochaetota also showed slight increases in abundance at LC500, but Proteobacteria abundance declined drastically. The population of methanogenic Euryarchaeota reduced continuously as the LC concentration increased. Overall, LC induced the core rumen microbiota to restructure in a dose-dependent manner, favoring Firmicutes over Proteobacteria and Bacteroidota.

#### 3.4.2 Genus level composition

**Figure 2:**
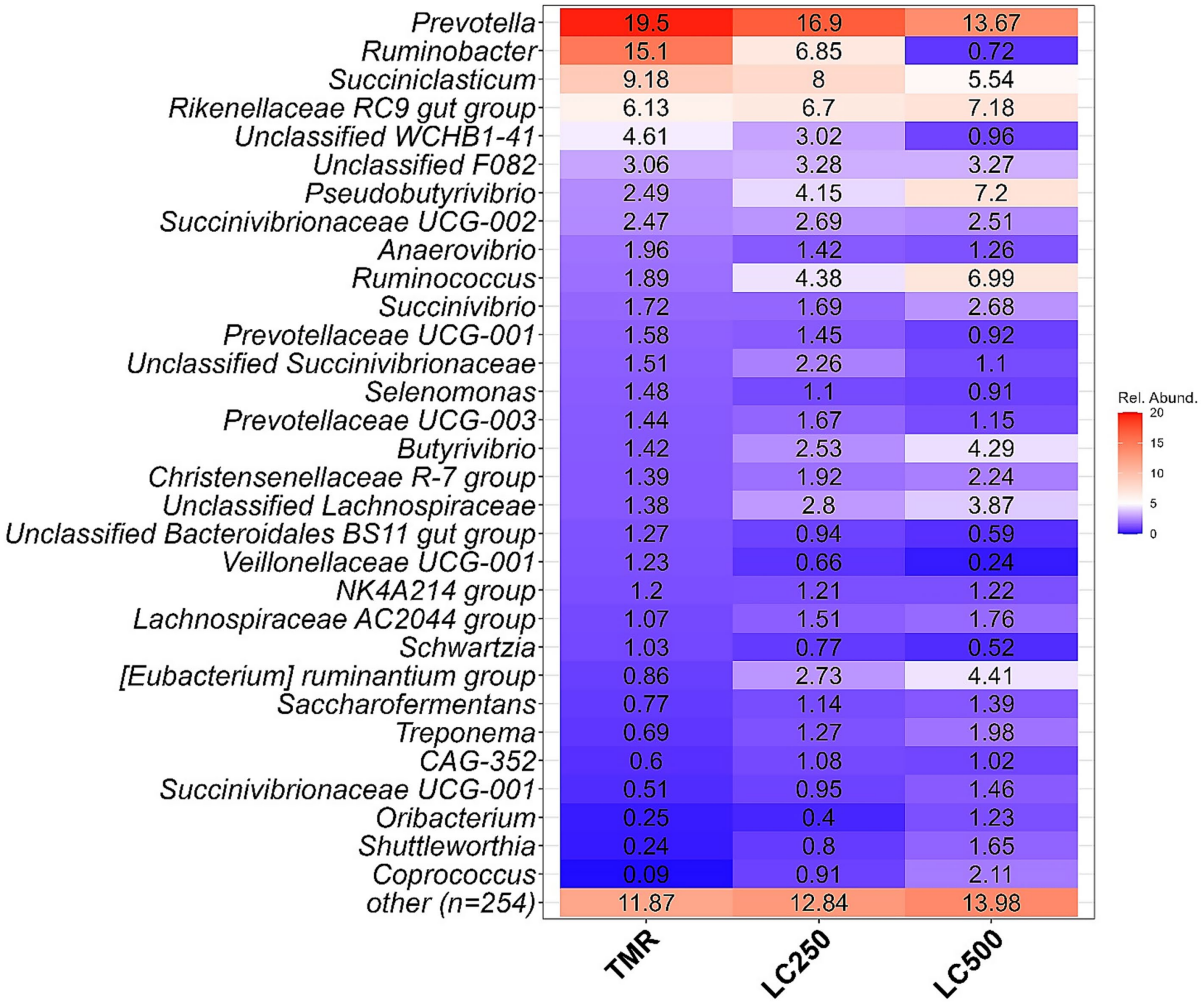
Relative abundance (%) of the 30 most abundant bacterial genera across dietary treatments. TMR (Total Mixed Ration; 0 μL/L LC), LC250 (TMR + 250 μL/L LC), and LC500 (TMR + 500 μL/L LC). Values represent mean relative abundance of each genus within each treatment group. Only the top 30 genera are shown; remaining low-abundance genera (<0.1% relative abundance each) were grouped as “Others.” Colour intensity reflects relative abundance as indicated in the legend.

L-carvone (LC) supplementation induced clear, dose-dependent shifts in the structure of the rumen bacterial community at the genus level (Figure 2; Table 5). *Prevotella* and *Ruminobacter* were the dominant genera in the TMR microbiota, and their abundance decreased significantly as the LC concentrations increased, with all three treatment groups differing significantly from one another (*p* < 0.001). Specifically, *Prevotella* decreased from 19.5% in TMR to 16.9% (LC250) and 13.67% (LC500), while *Ruminobacter* declined sharply from 15.1% (TMR) to 6.85% (LC250) and 0.72% (LC500). *Succiniclasticum* also declined overall (9.18 to 8.00 to 5.54%; *p* < 0.001), though the reduction was only significant between TMR and LC500, with LC250 not differing from either group. Several genera linked to butyrate production in Firmicutes increased significantly with LC supplementation, although the pattern of significance differed among taxa. *Butyrivibrio* and *[Eubacterium] ruminantium* group increased significantly across all three treatment groups, with TMR, LC250, and LC500 each differing significantly from one another (*p* < 0.001). *Butyrivibrio* increased from 1.42% (TMR) to 2.53% (LC250) and 4.29% (LC500), while *[Eubacterium] ruminantium* group increased from 0.86% (TMR) to 2.73% (LC250) and 4.41% (LC500). *Pseudobutyrivibrio*, and *Shuttleworthia* were significantly higher in both LC-supplemented groups relative to TMR (*p* < 0.001) but did not differ significantly between LC250 and LC500. *Pseudobutyrivibrio* increased from 2.49% in TMR to 4.15% (LC250) and 7.20% (LC500); and *Shuttleworthia* increased from 0.24 to 0.80 and 1.65%. *Oribacterium* increased significantly only at the highest inclusion level, with no significant difference between TMR and LC250 (0.25, 0.40, and 1.23% in TMR, LC250, and LC500, respectively; *p* < 0.001). In response to LC, fibrolytic genera also increased significantly. *Ruminococcus* rose from 1.89% (TMR) to 4.38% (LC250) and 6.99% (LC500), with all three treatment groups significantly different from one another (*p* < 0.001). The unclassified *Lachnospiraceae* members followed the same pattern, increasing from 1.38 to 2.80 and 3.87% with all treatment groups significantly different (*p* < 0.001), whereas the *Lachnospiraceae AC2044 group* increased significantly from TMR to LC250 (1.07–1.51%) but did not increase further at LC500 (1.76%; *p* < 0.001). In contrast, succinate- and propionate-producing genera were significantly reduced by LC supplementation, though the pattern of decline was not consistent across all genera. V*eillonellaceae UCG-001* declined significantly across all three treatment groups (1.23 to 0.66 to 0.24%; *p* < 0.001). *Selenomonas* declined overall from 1.48% (TMR) to 0.91% (LC500; *p* = 0.025), but LC250 (1.10%) was not significantly different from either endpoint. Within the Succinivibrionaceae family, results were mixed: *Succinivibrio* showed no significant difference among treatment groups despite a numerical increase from 1.72 to 2.68% (*p* = 0.533), whereas *Succinivibrionaceae UCG-001* increased significantly from TMR to LC500 (0.51–1.46%; *p* = 0.003), with LC250 (0.95%) intermediate and not significantly different from either group. Minor taxa categorized as “others” raised from 12.0% in TMR to 16.1% at LC500, indicating an expansion of taxonomic diversity under LC exposure (*p* = 0.002).

**Table 5:** Differential abundance analysis of top 30 bacterial genera across three dietary treatments.

Genus	^1^Dietary treatments			SEM	Adjusted *p*-value
	TMR	LC250	LC500		
*Prevotella*	19.5^a^	16.9^b^	13.67^c^	0.83	<0.001
*Ruminobacter*	15.1^a^	6.85^b^	0.72^c^	2.05	<0.001
*Succiniclasticum*	9.18^a^	8.00^a^	5.54^b^	0.95	<0.001
*Rikenellaceae RC9 gut group*	6.13^b^	6.70^ab^	7.18^a^	0.44	0.031
*Unclassified WCHB1-41*	4.61^a^	3.02^b^	0.96^c^	0.40	<0.001
*Unclassified F082*	3.06	3.28	3.27	0.42	0.504
*Pseudobutyrivibrio*	2.49^b^	4.15^a^	7.20^a^	1.29	<0.001
*Succinivibrionaceae UCG-002*	2.47	2.69	2.51	0.86	0.973
*Anaerovibrio*	1.96^a^	1.42^ab^	1.26^b^	0.42	0.034
*Ruminococcus*	1.89^c^	4.38^b^	6.99^a^	0.46	<0.001
*Succinivibrio*	1.72	1.69	2.68	0.63	0.533
*Prevotellaceae UCG-001*	1.58^a^	1.45^a^	0.92^b^	0.09	<0.001
*Unclassified Succinivibrionaceae*	1.51^b^	2.26^a^	1.10^b^	0.30	<0.001
*Selenomonas*	1.48^a^	1.10^ab^	0.91^b^	0.24	0.025
*Prevotellaceae UCG-003*	1.44^ab^	1.67^a^	1.15^b^	0.13	<0.001
*Butyrivibrio*	1.42^c^	2.53^b^	4.29^a^	0.58	<0.001
*Christensenellaceae R-7 group*	1.39^b^	1.92^ab^	2.24^a^	0.25	<0.001
*Unclassified Lachnospiraceae*	1.38^c^	2.80^b^	3.87^a^	0.21	<0.001
*Unclassified Bacteroidales BS11 gut grp*	1.27^a^	0.94^ab^	0.59^b^	0.23	<0.001
*Veillonellaceae UCG-001*	1.23^a^	0.66^b^	0.24^c^	0.12	<0.001
*NK4A214 group*	1.20	1.21	1.22	0.16	0.595
*Lachnospiraceae AC2044 group*	1.07^b^	1.51^a^	1.76^a^	0.21	<0.001
*Schwartzia*	1.03^a^	0.77^b^	0.52^b^	0.14	<0.001
*[Eubacterium] ruminantium group*	0.86^c^	2.73^b^	4.41^a^	0.43	<0.001
*Saccharofermentans*	0.77^b^	1.14^a^	1.39^a^	0.12	<0.001
*Treponema*	0.69^b^	1.27^ab^	1.98^a^	0.47	0.008
*CAG-352*	0.60	1.08	1.02	0.25	0.080
*Succinivibrionaceae UCG-001*	0.51^b^	0.95^ab^	1.46^a^	0.29	0.003
*Oribacterium*	0.25^b^	0.40^b^	1.23^a^	0.40	<0.001
*Shuttleworthia*	0.24^b^	0.80^a^	1.65^a^	0.30	<0.001
Others	12.0^b^	13.7^a^	16.1^a^	0.54	0.002

^1^TMR, total mixed ration = (0 μL/L of L-carvone); LC250 = (TMR + 250 μL/L of L-carvone); LC500 = (TMR + 500 μL/L of L-carvone). ^a–c^Means with different superscripts in a row differ significantly (*p* < 0.05). *p-*values were adjusted for multiple testing using the Benjamini–Hochberg false discovery rate (FDR) procedure. Only the 30 most abundant genera are shown; remaining low-abundance genera (<0.1% relative abundance) were grouped as “Others”.

#### 3.4.3 Alpha diversity

**Figure 3:**
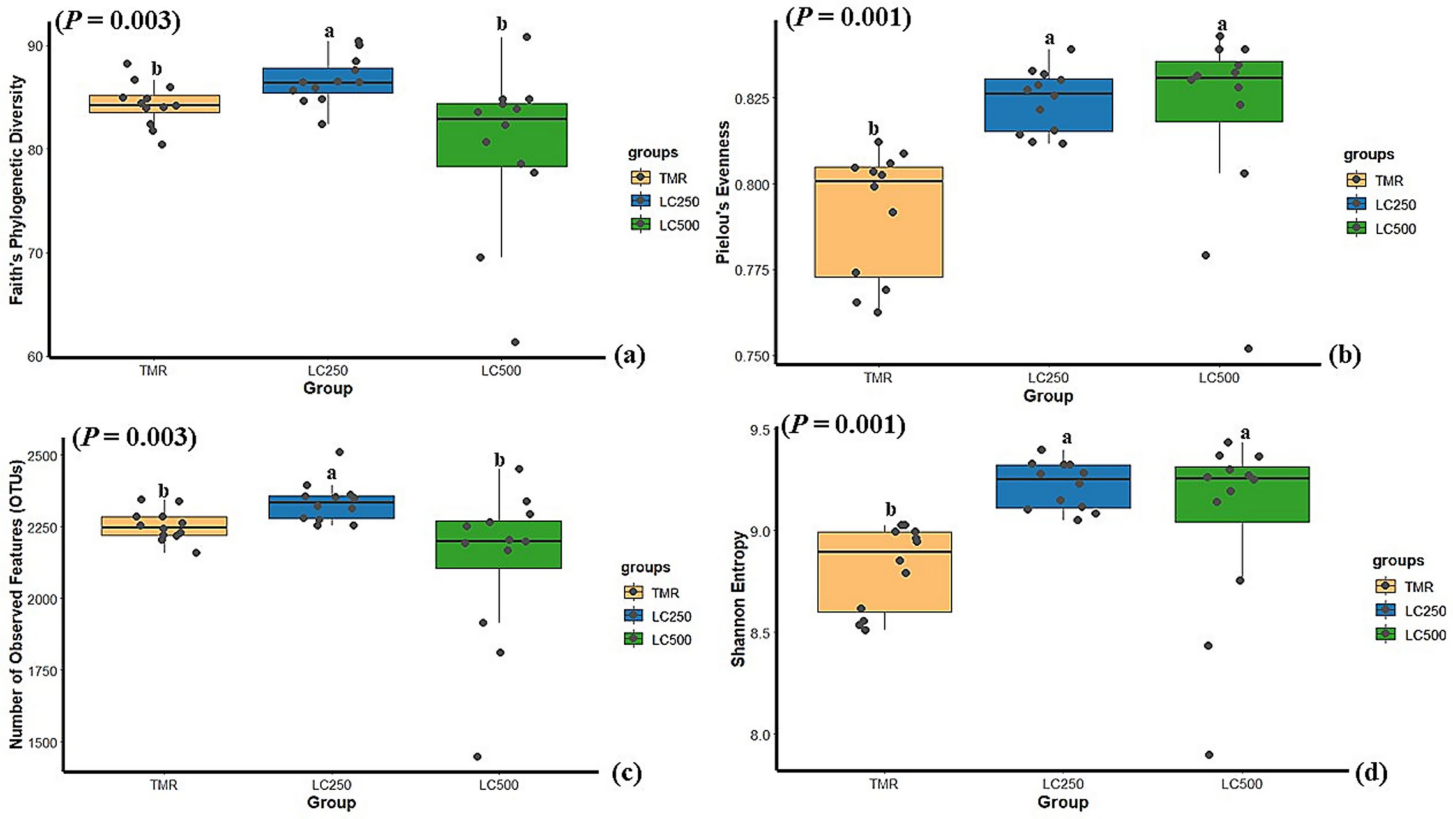
Microbial diversity metrics across treatment groups; TMR (Total Mixed Ration; 0 μL/L LC), LC250 (TMR + 250 μL/L LC), and LC500 (TMR + 500 μL/L LC). Subplots show: **(a)** Faith’s Phylogenetic Diversity, **(b)** Pielou’s Evenness, **(c)** Number of Observed Features (OTUs), and **(d)** Shannon Entropy. Each box plot represents the distribution of the metric within a treatment group. Different letters indicate significant differences among treatments based on pairwise comparisons using adjusted *p*-values (*p* < 0.05).

LC supplementation had a significant impact on alpha diversity metrics (Figure 3). LC250 displayed the highest Faith’s Phylogenetic Diversity (PD) and observed OTUs as compared to TMR and LC500 (*p =* 0.003). Shannon entropy and Pielou’s evenness were lowest in TMR but increased significantly under both LC treatments (*p =* 0.001).

#### 3.4.4 Beta diversity

**Figure 4:**
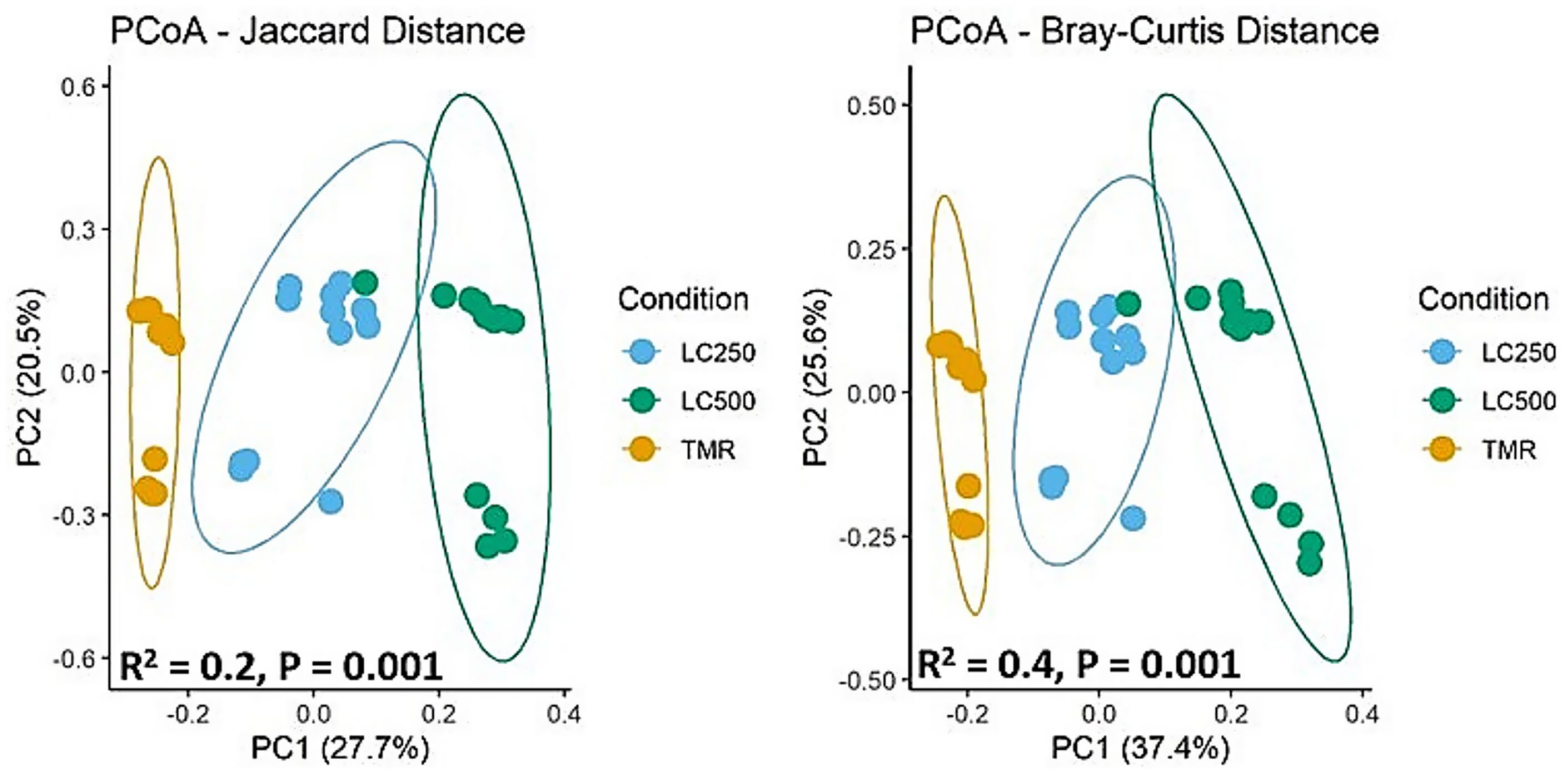
Principal Coordinate Analysis (PCoA) of microbial communities based on Jaccard and Bray–Curtis distances across treatment groups. Multivariate effects of treatments on beta diversity were evaluated by non-parametric permutational multivariate analysis of variance (PERMANOVA).

Bray–Curtis and Jaccard dissimilarities showed prominent clustering among treatments (Figure 4). TMR made a compact cluster, LC250 was in an intermediate position, and LC500 were substantially separated, accounting for 27.7% (PC1) + 20.5% (PC2) of Jaccard variance. PC1 and PC2 of Bray–Curtis accounts for 37.4 and 25.6% of the variance, respectively. These compositional differences were statistically supported by PERMANOVA (Jaccard: *R*^2^ = 0.20, *p* < 0.001; Bray–Curtis: *R*^2^ = 0.40, *p* < 0.001).

#### 3.4.5 Correlation between volatile fatty acids, methane production, and the relative abundance of dominant rumen bacterial genera

**Figure 5:**
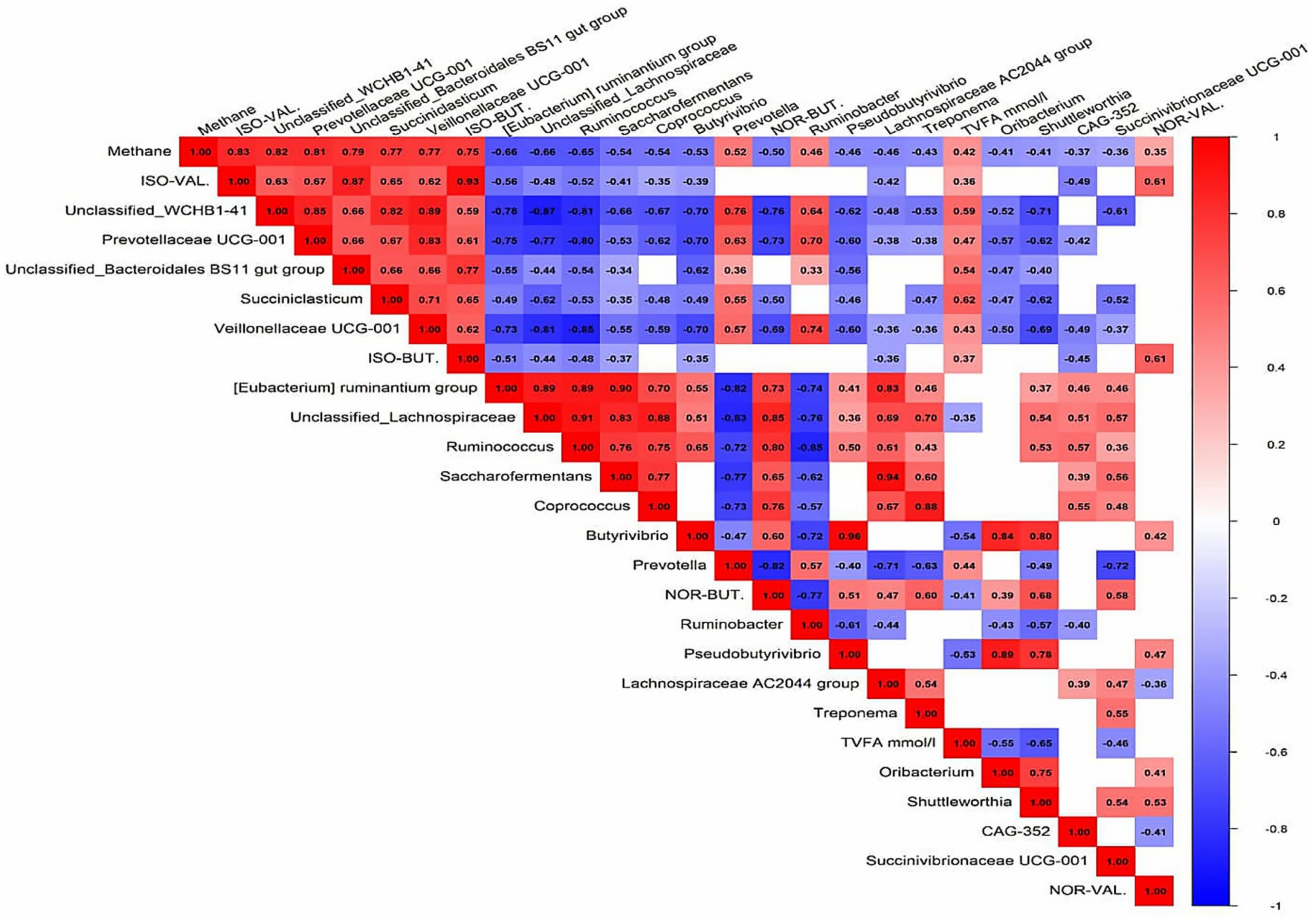
Pearson’s correlation heatmap showing relationships between methane production, volatile fatty acids, and the relative abundance of dominant rumen bacterial genera. Red and blue colours indicate positive and negative correlations, respectively, with colour intensity reflecting the strength of the correlation. Correlation coefficients (*r*) are shown within each cell.

Clear associations between CH_4_ production, VFA profiles, and the relative abundance of dominant rumen bacterial genera were identified by Pearson’s correlation analysis (Figure 5). Branched-chain fatty acids, such as iso-valerate and iso-butyrate, and taxa related to Bacteroidota such as *Prevotella,* were positively correlated with CH_4_ production. In contrast, CH_4_ production showed strong negative correlations with several Firmicutes genera, including *Butyrivibrio*, *Pseudobutyrivibrio*, and *Ruminococcus*, which are recognized for their roles in butyrate production. Furthermore, the relative abundance of butyrate-producing genera was positively associated with butyrate concentration, whereas branched-chain VFA’s were positively correlated with *Prevotella*.

## 4 Discussion

### 4.1 Gas production, methane, IVDMD, VFA, pH, and ammonia-nitrogen

The LC supplementation altered rumen fermentation, digestibility, and microbial community composition, with more pronounced effects observed at the higher inclusion level. The reduction in total gas and CH_4_ production with LC500 suggests a strong inhibitory action on rumen microbial activity. A decline in gas output typically reflects reduced fermentation of dietary organic matter (30). The potential of EOs to mitigate CH_4_ production has been extensively investigated; however, their efficacy largely depends on their phytochemical composition and the bioactive compounds present in them (31, 32).

L-carvone (LC), a monoterpene, likely increases membrane fluidity and depletes microbial ATP and destabilize microbial membranes, interfere with enzymatic activity, and act as a proton exchanger (33), thereby reducing microbial activity and ultimately decreasing CH_4_ production. Euryarchaeota, a phylum belonging to the archaea community, showed low relative abundance and reduced with increasing supplementation of LC, corresponding to the CH_4_ reduction in LC treatments. These findings suggest that the relative abundance of archaea may have a greater impact on ruminal CH_4_ production than the microbial community structure (34).

Moreover, the current trial demonstrated a 15.1% reduction in digestibility in the LC500 group compared to the TMR. This reduction aligns with the observed decline in primary carbohydrate-degrading taxa, particularly *Prevotella* and *Ruminobacter*, which are central contributors to hemicellulose degradation and peptide metabolism in the rumen (35). Suppressing these genera most likely lowered fibrolytic and saccharolytic activity, resulting in lesser nutrient availability for microbial fermentation and explained the lower IVDMD under LC500. In contrast, LC250 digestibility values were comparable to the TMR, suggesting that LC at lower dose can influence fermentation without negatively impacting substrate degradation. The VFA shifts observed further support a dose-dependent modulation of microbial pathways. The reduction in total VFA at LC500, is in line with the decreased microbial fermentation activity. The LC inhibited succinate-to-propionate pathways, as evidenced by the reduced propionate concentration and decline in propionate-associated genera (*Ruminobacter*, *Selenomonas*, *Veillonellaceae_UCG-001*) in LC500.

On the other hand, butyrate concentration enhanced significantly, accompanied by substantial enrichment of butyrogenic Firmicutes, such as *Pseudobutyrivibrio*, *Butyrivibrio*, *Coprococcus*, and *[Eubacterium] ruminantium*. This association was further supported by correlation analysis, which showed that these genera were negatively correlated with CH_4_ production, highlighting their role in shifting rumen fermentation toward butyrate formation under LC supplementation. Furthermore, butyrate functions as an essential energy source for epithelial cells and influences nutrient utilization and rumen function (36). The pH displayed numerically high value at LC500, presumably attributable to the decline in VFA production. The EOs and their bioactive constituents may diminish ruminal protein breakdown and ammonia concentrations (37). They can suppress hyper-ammonia-producing bacteria (HAB) and limit amino acid fermentation, thereby lowering ammonia output (38). In line with previous studies reporting that EOs reduce ruminal NH_3_-N concentrations, the tendency toward lower NH_3_-N concentration at the higher LC inclusion level may indicate a partial suppression of ruminal protein degradation and amino acid deamination (38–40).

### 4.2 Rumen microbial community

To our knowledge, no previous studies have reported on the effects of LC on bacterial community structure in ruminants, restricting a comprehensive understanding of their impact on ruminal bacterial communities. The current study used 16S rRNA sequencing to compare the impact of LC on ruminal microbial composition. Firmicutes, Bacteroidetes, and Proteobacteria were the dominant bacteria in this study, consistent with the earlier findings (35, 41, 42). Gram-positive bacteria are generally considered more susceptible to EOs than gram-negative bacteria, owing to the absence of a protective outer membrane (43).

The decline in gram-negative phylum Bacteroidota and increase in gram-positive phylum Firmicutes across LC treatments reflect a selective antimicrobial effect (44), consistent with previous reports where terpenoid compounds suppressed gram-negative taxa more than gram-positive populations (45, 46). Although gram-negative bacteria are generally less susceptible to EOs due to the lipopolysaccharide’s outer membrane, the observed reduction in Bacteriodata suggests that LC exhibited antimicrobial effect across both gram-negative and gram-positive microbes (14, 47). Furthermore, we noted that *Prevotella* was the predominant genus across all treatments, consistent with some recent *in vivo* studies reporting its prevalence in rumen microbial communities (35, 42, 48). However, the substantial decline observed in *Prevotella*, *Ruminobacter,* and *Succiniclasticum* when LC concentration increased and these genera plays a key role in the succinate-propionate pathway in the rumen. Thus, the decrease in *Prevotella*, *Ruminobacter,* and *Succiniclasticum* abundance with increasing LC likely contributed to the lower propionate concentrations through reduced succinate production and utilization (49). *Rikenellaceae RC9 gut group*, a gram-negative member of the Bacteroidota phylum are associated with primary or secondary carbohydrate degradation (50), showed increased abundance under LC treatment. Firmicutes, particularly fibrolytic and butyrate-producing genera including *Pseudobutyrivibrio*, *Butyrivibrio*, and *Ruminococcus*, propagated concurrently consistent with the findings of Patra and Yu (43), who reported increased Firmicutes abundance under EOs exposure. The shift reflects a selective environment that favours butyrogenic Firmicutes and is consistent with the elevated butyrate level in LC500.

Alpha-diversity profiles demonstrated a non-linear response; for example, LC250 increased PD and microbiological richness, whereas a high dose LC500 decreased richness while maintaining high evenness. This behaviour is characteristic of bioactive phytochemicals, which initially promote community restructuring and niche redistribution but then supress sensitive microbes at high levels (51). Correlation analysis further strengthened the mechanistic interpretation of LC effects on rumen fermentation. CH_4_ production was strongly and positively associated with branched-chain fatty acids, particularly iso-valerate, as well as with *Prevotella* abundance, indicating that reductions in these fermentation products and taxa under LC supplementation coincided with CH_4_ mitigation and reflected broader microbial shifts associated with altered fermentation pathways. In contrast, CH_4_ emission was negatively correlated with butyrate-producing Firmicutes, including *Butyrivibrio, Pseudobutyrivibrio, and Ruminococcus,* which were enriched in LC500. These relationships support the role of LC in reshaping microbial interactions linked to CH_4_ formation. Overall, these findings indicate that LC modulates rumen fermentation through selective microbial restructuring. While LC500 achieved the highest reduction in CH_4_ production, this response was accompanied by lower IVDMD and total VFA concentration, suggesting a broader inhibition of rumen fermentation activity. In contrast, LC250 maintained IVDMD and total VFA concertation without negatively affecting fermentation, while exhibiting a numerical decline in CH_4_ production and modest shifts in microbial composition.

## 5 Conclusion

Supplementation of LC at 500 μL/L reduced CH_4_ production by 28% *in vitro*; however, this reduction was accompanied by decreased total gas production, lower TVFA concentration and reduced IVDMD, indicating impaired overall fermentation at LC500. Increasing LC concentration altered rumen microbial composition, including a decline in *Prevotella* and *Ruminobacter*, along with a consistent reduction in methanogenic Euryarchaeota, supporting a microbial-mediated mechanism of CH_4_ suppression. These findings indicate that LC has potential as a CH_4_ mitigating feed additive; however, excessive inclusion may negatively affect fermentation efficiency and digestibility. Therefore, optimization of LC dosage and *in vivo* validation are required to achieve effective CH_4_ mitigation without compromising nutrient utilization and animal performance.

## Data Availability

The original contributions presented in the study are publicly available. This data can be found here: The 16S rRNA gene sequencing data presented in this study are deposited in the NCBI BioProject repository under accession number PRJNA1499012.

## Footnotes

1
https://github.com/genpat-it

2
https://nf-co.re/ampliseq/2.12.0
